# Optimizing Mental Stress Detection via Heart Rate Variability Feature Selection

**DOI:** 10.3390/s25134154

**Published:** 2025-07-03

**Authors:** Mohsen Behradfar, Shotabdi Roy, Joseph Nuamah

**Affiliations:** School of Industrial Engineering and Management, Oklahoma State University, Stillwater, OK 74078, USA; mohsen.behradfar@okstate.edu (M.B.); shotabdi.roy@okstate.edu (S.R.)

**Keywords:** electrocardiogram, feature selection, heart rate variability, mental stress detection, machine learning, recursive feature elimination

## Abstract

The increasing prevalence of stress-related disorders necessitates accurate and efficient detection methods for timely intervention. This study explored the potential of heart rate variability as a biomarker for detecting mental stress using a publicly available dataset. A total of 93 heart rate variability features extracted from electrocardiogram signals were analyzed to differentiate stress from non-stress conditions. Our methodology involved data preprocessing, feature computation, and three feature selection strategies—filter-based, wrapper, and embedded—to identify the most relevant heart rate variability features. By leveraging Recursive Feature Elimination combined with Nested Leave-One-Subject-Out Cross-Validation, we achieved a peak F1 score of 0.76. The results demonstrate that two heart rate variability features—the median absolute deviation of the RR intervals (the time elapsed between consecutive R-waves on an electrocardiogram), which is normalized by the median, and the normalized low frequency power—consistently distinguished the stress states across multiple classifiers. To assess the robustness and generalizability of our best-performing model, we evaluated it on a completely unseen dataset, which resulted in an average F1 score of 0.63. These findings emphasize the value of targeted feature selection in optimizing stress detection models, particularly when handling high-dimensional datasets with potentially redundant features. This study contributes to the development of efficient stress monitoring systems, paving the way for improved mental health assessment and intervention.

## 1. Introduction

Mental stress is a significant global health concern, contributing to cardiovascular diseases, metabolic disorders, and neuropsychiatric conditions, such as anxiety and depression. Chronic stress disrupts autonomic nervous system (ANS) regulation, leading to heightened sympathetic activity, reduced parasympathetic function, and increased health risks [1,2]. Stress-related impairments in cognitive function and decision making have been linked to dysfunctions in the prefrontal cortex, negatively affecting workplace productivity and increasing mental health disorders [3]. The World Health Organization has recognized stress-related illnesses as a leading cause of disability worldwide, emphasizing the need for objective, real-time stress monitoring systems [4].

Heart rate variability (HRV), derived from electrocardiogram (ECG) signals or photoplethysmogram, has emerged as a robust physiological biomarker for assessing stress due to its direct association with ANS activity and emotional regulation [5,6]. HRV measures fluctuations in the heart rate over time, reflecting the interplay between sympathetic and parasympathetic influences on cardiac function, making it a valuable tool for stress classification [7,8]. HRV-based stress detection has been widely explored in various domains, including occupational stress assessment [9], clinical diagnostics [10], and sports performance monitoring [11]. However, the effectiveness of HRV-based stress classification depends on the selection of relevant features, as high-dimensional HRV datasets often contain redundant or irrelevant information, leading to decreased model interpretability and increased computational complexity [12].

Machine learning (ML) techniques have been extensively used to classify the stress states from HRV signals [13]. Recently, deep learning approaches, such as Convolutional Neural Networks [14] and Recurrent Neural Networks (RNNs) [15], have demonstrated high classification accuracy. However, deep learning models often require extensive labeled datasets and substantial computational resources, making traditional ML models preferable in real-time, low-power applications [16].

Feature selection plays a crucial role in HRV-based stress detection by reducing dimensionality and improving model generalizability. Feature selection methods can be broadly categorized into filter-based, wrapper-based, and embedded approaches. Filter-based methods, such as Minimum Redundancy Maximum Relevance (mRMR), rank features based on their statistical relevance while minimizing redundancy [17]. Wrapper-based techniques, such as Recursive Feature Elimination (RFE), iteratively evaluate feature subsets by training a classifier and eliminating the least informative features [18]. Embedded methods, such as Least Absolute Shrinkage and Selection Operator (LASSO), integrate feature selection into the model training process using L1 regularization to enforce sparsity [19].

Several studies have demonstrated the importance of feature selection in HRV-based classification. Byun et al. [20] showed that entropy-based feature selection improves stress classification accuracy in distinguishing individuals with Major Depressive Disorder. Shikha et al. [21] applied explainable AI techniques to optimize HRV-based stress classification in wearable biosensors, enhancing model interpretability. Despite these advancements, selecting the most relevant HRV features for stress detection remains an open research problem, requiring further evaluation across multiple classifiers and feature selection methods.

An additional challenge in HRV-based ML models is model validation. Many prior studies have relied on k-fold cross-validation, which may introduce data leakage due to intra-subject correlations in physiological datasets [22]. Leave-One-Subject-Out Cross-Validation (LOSO-CV) has emerged as a preferred approach for HRV-based classification, ensuring that models generalize to unseen individuals by holding out one subject’s data per training iteration [23]. Nested LOSO-CV further enhances model robustness by incorporating an inner-loop validation process for hyperparameter tuning, preventing information leakage and improving reproducibility [24].

The objective of the present study was to evaluate three different feature selection techniques—mRMR, RFE, and LASSO—and to apply them to 93 HRV features to identify the optimal subset that best distinguishes stress states from non-stress states. This paper is organized as follows: Section 2 outlines the methodology, including the feature selection processes and machine learning models. Section 3 presents the results of the feature selection and classification experiments. Section 4 discusses the implications of the findings, comparing them to prior work. Finally, Section 6 concludes this paper by summarizing the main contributions and suggesting potential areas for future research.

## 2. Materials and Methods

### 2.1. Dataset and Data Preprocessing

In this study, we utilized ECG data from the publicly available WESAD dataset, which was collected by researchers in Germany [25]. This dataset comprises various physiological signals, including electrocardiogram, electrodermal activity, electromyogram, respiration, temperature, and motion data. The ECG data were recorded at a sampling rate of 700 Hz using a chest-worn device (RespiBAN, PLUX Wireless Biosignals S.A., Lisbon, Portugal). The dataset includes recordings from 15 participants, consisting of 12 males and 3 females, with an average age of 27.5 ± 2.4 years. Participants were selected based on specific criteria, excluding individuals who were pregnant, heavy smokers, or those with mental disorders or cardiovascular diseases. Each participant’s data were also linked to self-reported experiences corresponding to four different experimental conditions: Baseline, Amusement, Stress, and Meditation. Approximately 36 min of data were collected per participant.

**Figure 1:**
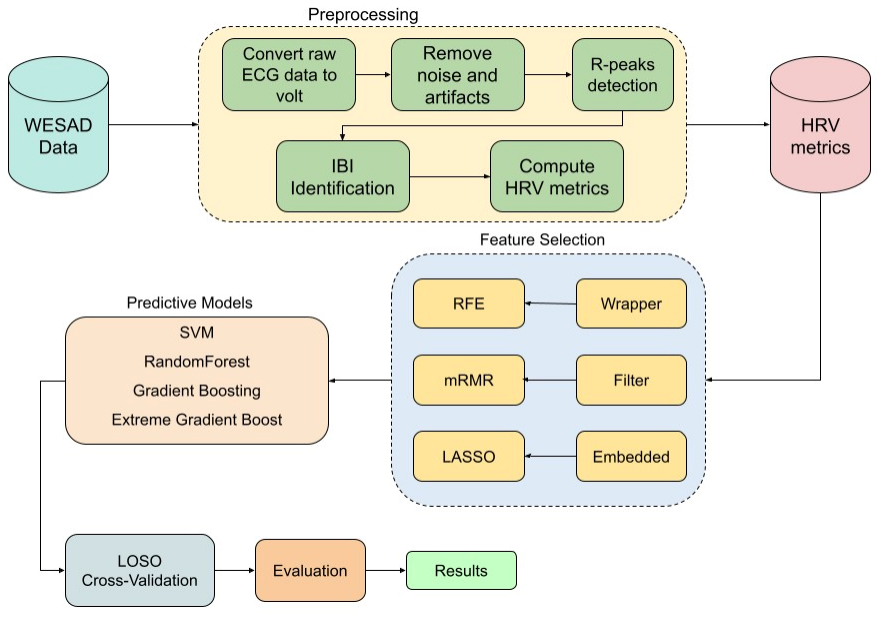
Flow diagram of the methodology.

The ECG data used in this study underwent a comprehensive preprocessing pipeline using the NeuroKit2 library [26] to extract 93 HRV features across multiple domains. Initially, the raw ECG signal was converted to volts to ensure accurate amplitude representation, and any missing values were dropped to maintain data integrity. The signal was then cleaned using NeuroKit2’s *ecg-clean* function, which applies built-in filtering techniques to remove noise and artifacts, enhancing the reliability of subsequent analyses. To detect R-peaks, we employed the Pan–Tompkins algorithm, a well-established method for QRS complex detection in ECG signals [27]. The ECG data were segmented into fixed-length 1 min windows to ensure consistency across subjects and conditions. R-peaks were identified within each segment, and corresponding inter-beat intervals (IBIs) were computed to serve as the foundation for HRV analysis. These IBIs were then passed to the *hrv* function of the NeuroKit2 package for comprehensive HRV feature extraction. NeuroKit2 internally applies preprocessing routines, such as signal quality checks and interpolation, to enhance the reliability of HRV features. In total, 93 HRV features were extracted, spanning time-domain, frequency-domain, and nonlinear measures. Detailed descriptions of all HRV features and their corresponding abbreviations are provided in the Abbreviations section at the end of this paper. After extracting HRV features, each sample was assigned a label corresponding to its respective experimental condition, ensuring the dataset was properly structured for subsequent classification tasks. To binarize the problem, and since we are only interested in distinguishing between stress and non-stress conditions, we labeled the stress condition as ‘stress’ and grouped all other conditions under ‘non-stress.’ The entire workflow is illustrated in the flow diagram in Figure 1.

To assess the generalizability of our proposed model beyond the primary dataset, we conducted an external validation using an independent dataset obtained from PhysioNet titled “A Wearable Exam Stress Dataset for Predicting Cognitive Performance in Real-World Settings [28].” This publicly available dataset comprises physiological recordings from 10 university students, and they were captured using the Empatica E4 wearable device during three academic examination sessions: Midterm 1, Midterm 2, and the Final exam. The midterm sessions each lasted approximately 1.5 h, while the final exam extended over a 3 h period. Among the recorded physiological signals, the IBI data were extracted for analysis. We applied the same preprocessing and feature extraction procedures used in our primary dataset to ensure methodological consistency. For binary classification purposes, the Midterm 1 session was designated as the non-stress (Baseline) condition, whereas the Final exam was considered the high-stress condition. This external validation enabled us to evaluate the performance of our best-performing model on completely unseen data, thereby offering critical insight into its robustness, transferability, and potential application in real-world stress detection contexts.

### 2.2. Feature Selection Methods

Feature selection is a crucial step in ECG-based ML models to optimize classification accuracy and computational efficiency. In this study, we employed three feature selection approaches: filter-based, wrapper, and embedded methods. Specifically, we utilized mRMR (filter-based), RFE (wrapper), and embedded methods, including LASSO Regression (L1 Regularization), and tree-based models (Random Forest (RF), Extreme Gradient Boosting (XGB), and Gradient Boosting (GB)). These methods have been widely used in ECG signal analysis, demonstrating their effectiveness in enhancing predictive performance by selecting the most relevant features while minimizing redundancy [12,29,30].

#### 2.2.1. Filter-Based Approach

The mRMR method is a filter-based technique that selects features by maximizing their relevance to the target variable while minimizing the redundancy among selected features. This is achieved by ranking features based on mutual information, ensuring that selected features are both informative and non-redundant. The relevance *D* and redundancy *R* are defined as

(1) $$D=\frac{1}{\left|S\right|}\sum_{f_{i}\in S}I\left(f_{i},y\right),$$

(2) $$R=\frac{1}{{\left|S\right|}^{2}}\sum_{f_{i},f_{j}\in S}I\left(f_{i},f_{j}\right),$$

where $I(f_{i},y)$ represents the mutual information between feature $f_{i}$ and the target variable *y*, and $I(f_{i},f_{j})$ denotes the mutual information between selected features. The objective is to maximize D−R to select the most discriminative yet independent features [12,17].

To implement this method, we experimented with three different feature set sizes, selecting the top 10, 20, and 30 features based on the mRMR ranking. This approach allowed us to evaluate the impact of the feature subset size on classification performance and to determine the optimal number of selected features. Once the feature selection process was completed, we applied four machine learning classifiers—RF, Support Vector Machine (SVM), XGB, and Gradient Boosting—to assess their predictive capabilities on the selected feature subsets. The performance of each classifier was evaluated to identify the most effective combination of feature selection and classification for stress detection.

#### 2.2.2. Wrapper-Based Approach

The RFE method is a wrapper-based technique that iteratively removes the least important features based on their impact on model performance. A base classifier, such as RF, is trained multiple times, and features are ranked according to their importance scores. The least informative features are eliminated at each iteration until the optimal subset is obtained [31,32].

To enhance the robustness of feature selection, Recursive Feature Elimination with Cross-Validation (RFE-CV) was implemented within the nested LOSO-CV framework. In each inner loop iteration, RFE was applied to the training set using four different classifiers—RF, SVM, XGB, and GB—as base estimators. The optimal number of features was determined by maximizing the F1 score, and the selected feature subset was then evaluated in the outer loop to ensure its generalizability across unseen subjects.

#### 2.2.3. Embedded Approach

In this study, we employed embedded methods, i.e., LASSO Regression (L1 Regularization), and tree-based models (RF, XGB, and GB) to identify the most informative HRV features. LASSO Regression (L1 Regularization) applies $L_{1}$ regularization to eliminate irrelevant features by penalizing their coefficients [19]. The LASSO objective function is given by

(3) $$\min_{\beta }\sum_{i=1}^{n}{\left(y_{i}-\beta_{0}-\sum_{j=1}^{p}\beta_{j}x_{ij}\right)}^{2}+\lambda \sum_{j=1}^{p}\left|\beta_{j}\right|,$$

where λ controls the degree of regularization. A higher value of λ forces more feature coefficients to zero, reducing the number of selected features while retaining those with the highest predictive power. The optimal subset of HRV features was determined by applying this method, selecting features that consistently improved classification performance. The selected feature sets were evaluated using four different classifiers—RF, SVM, XGB, and GB—with performance assessed based on the F1 score.

Tree-based feature selection methods are widely used for evaluating feature relevance by leveraging decision tree structures to determine the importance of each feature in predictive modeling. These methods assign importance scores to features based on their contribution to reducing impurity or improving model performance. A key advantage of tree-based methods is their ability to handle high-dimensional data while capturing complex feature interactions. They can be applied in ensemble models, such as RF, XGB, and GB, which aggregate multiple decision trees to improve feature ranking stability and enhance predictive performance.

RF determines feature relevance by evaluating the contribution of each feature to the overall model performance. Features that have a greater impact on the model’s predictive ability are assigned higher importance scores, while features with minimal contribution are excluded from the final feature subset [33].

GB and XGB assess feature importance by measuring the frequency and quality of splits involving a given feature during training. GB assigns importance based on the cumulative reduction in loss (e.g., mean squared error for regression and log-loss for classification) attributed to each feature. XGB extends GB by incorporating additional regularization techniques, such as L1/L2 penalties, and by employing a more efficient split-finding algorithm, leading to more robust feature selection [34].

### 2.3. Predictive Modeling and Cross-Validation Framework

Four machine learning classifiers were employed to analyze the selected features and classify emotional states. RF, an ensemble learning method based on decision trees, was utilized to enhance predictive performance by reducing variance through bootstrap aggregation. SVM was implemented as a kernel-based classifier capable of identifying an optimal hyperplane for feature separation in high-dimensional spaces. GB was applied as an iterative boosting technique that sequentially combines weak learners to minimize classification errors. Additionally, XGB, an advanced variant of GB, was incorporated to further optimize efficiency and introduce regularization mechanisms for improved model generalization.

To ensure robust and unbiased evaluation of model performance, a nested LOSO-CV framework was employed. This approach is particularly suited for subject-independent validation, which is critical when models are intended to generalize across individuals rather than overfit to idiosyncrasies of specific participants.

In the outer loop of the LOSO-CV, data from one subject were held out as an independent test set, while the model was trained on data from all the remaining subjects. This process was repeated iteratively until every subject had served once as the test subject. This exhaustive, rotation-based strategy simulates real-world deployment scenarios where models encounter entirely unseen individuals.

Within each outer-loop training fold, an inner LOSO-CV loop was applied exclusively to the training data. This inner loop was used to perform hyperparameter tuning and feature selection, ensuring that no information from the held-out test subject influenced the feature selection or model optimization process. Such a nested design is essential for avoiding information leakage, which can lead to overestimated performance metrics if hyperparameters or features are inadvertently tuned using knowledge of the test set.

The nested LOSO-CV thus serves two key purposes: (1) it evaluates the model’s generalizability across subjects in the outer loop, and (2) it ensures unbiased model optimization in the inner loop. By separating the test data from every stage of training and tuning, this strategy provides a reliable and rigorous estimate of real-world model performance in subject-independent contexts, such as biomedical or behavioral signal classification.

#### Hyperparameter Optimization and Evaluation

Hyperparameter tuning was performed using grid search on the inner loop, optimizing hyperparameters for our predictive models. The final model performance was assessed using the F1 score, a balanced metric between precision and recall [35]:

(4) $$F1\;score=2\times (\frac{\mathrm{Precision}\times \mathrm{Recall}}{\mathrm{Precision}+\mathrm{Recall}}),$$

where precision and recall are defined as [35]

(5) $$\mathrm{Precision}=\frac{\mathrm{True}\mathrm{Positive}}{\mathrm{True}\mathrm{Positive}+\mathrm{False}\mathrm{Positive}},$$

(6) $$\mathrm{Recall}=\frac{\mathrm{True}\mathrm{Positive}}{\mathrm{True}\mathrm{Positive}+\mathrm{False}\mathrm{Negative}}.$$

## 3. Results

The performance of the various feature selection methods (filter-based, wrapper-based, and embedded) across four different ML classifiers—RF, SVM, XGB, and GB—was evaluated using the F1 score. The models were tested using the LOSO-CV framework to assess their generalization performance. The findings are detailed in their respective subsections.

### 3.1. Filter-Based Approach

#### 3.1.1. mRMR 10 Features

In the initial evaluation, using mRMR with 10 features, the goal was to examine how effectively a compact, information-rich subset could sustain classification performance. The selected features (listed in Table 1) were chosen for their high relevance to the target class and low inter-feature redundancy, aligning with mRMR’s core objective of enhancing generalization through efficient representation.

**Figure 2:**
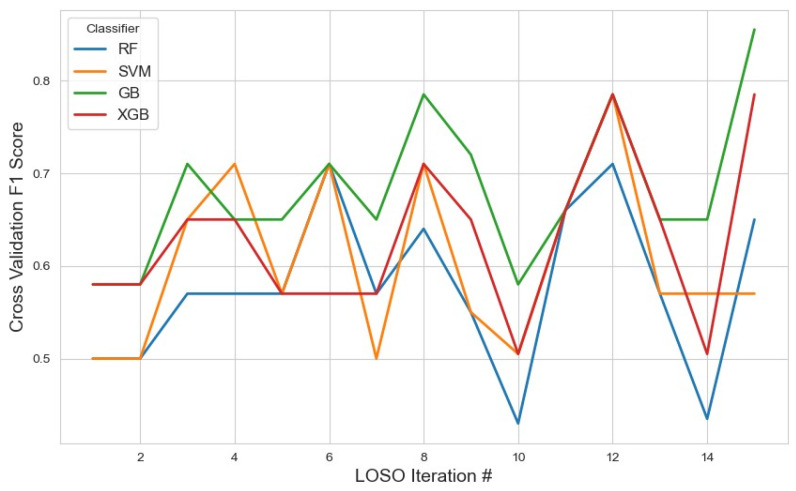
Performance metrics of the models across iterations using the top 10 features selected by the mRMR method. The plot illustrates how the classification performance evolved during cross-validation, highlighting model stability and feature selection impact.

Classifier performance on this subset was assessed using the average F1 score, with the results visualized in Figure 2. GB achieved the highest average F1 score of 0.67 (95% CI: 0.62, 0.72), indicating relatively stronger performance when leveraging a smaller but focused feature space. While this result suggests GB’s adaptability, the modest F1 score also implies that the 10-feature configuration may not fully capture the underlying complexity of the task.

XGB and SVM followed closely with scores of 0.62 and 0.61, respectively. These results suggest that, despite dimensionality reduction, the feature set retained core discriminatory power, especially for models like SVM that can benefit from well-separated feature distributions. RF performed the lowest at 0.57, potentially due to reduced ensemble diversity from fewer input variables.

**Figure 3:**
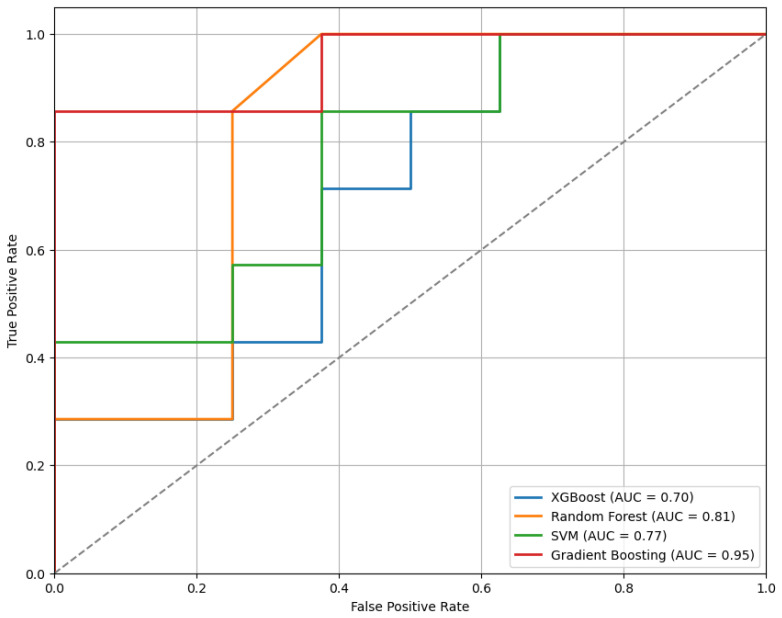
The ROC curves for the classifiers trained on the top 10 features selected by the mRMR method, illustrating comparative model performance.

Figure 3 presents the ROC curves for the models trained using the top 10 features selected by the mRMR method. As illustrated, the GB classifier achieves the highest performance among the evaluated models, with an Area Under the Curve (AUC) of 0.95, indicating strong discriminative ability.

#### 3.1.2. mRMR 20 Features

**Figure 4:**
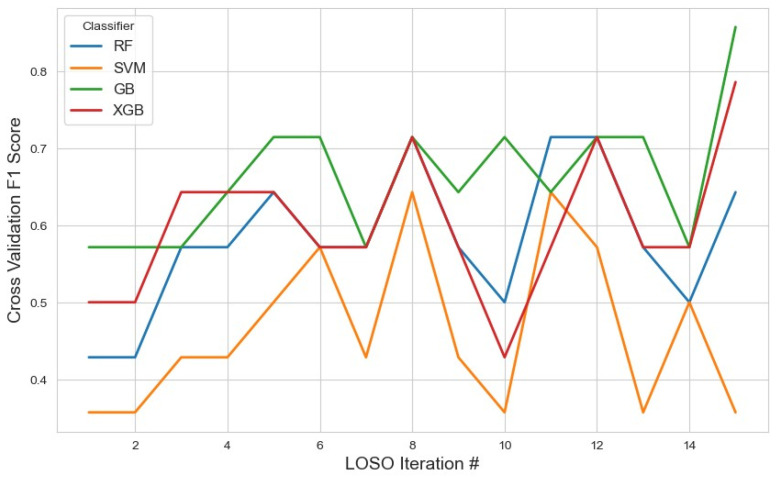
The model performance across iterations using the top 20 features selected by the mRMR method. This figure demonstrates how the classification metrics varied during cross-validation, reflecting the influence of feature selection on model consistency.

Expanding to 20 features (Figure 4) allowed mRMR to retain a broader subset of variables, potentially encompassing more nuanced patterns while still minimizing redundancy. The selected features are detailed in Table 1.

In this setting, GB again showed the highest average F1 score of 0.66 (95% CI: 0.61, 0.70). Although slightly lower than the 10-feature configuration, this suggests stable performance, possibly due to a balance between increased feature diversity and model complexity. The decline in performance might also hint at some redundancy or noise entering the feature space as dimensionality increased.

XGB remained consistent with a moderate score of 0.60, and RF followed at 0.58, indicating that these ensemble models continued to benefit from the structured feature selection, though gains were marginal. SVM, however, experienced a marked drop to 0.46, suggesting that kernel-based models may be more sensitive to feature redundancy or suffer from the curse of dimensionality, even in moderately expanded feature spaces.

**Figure 5:**
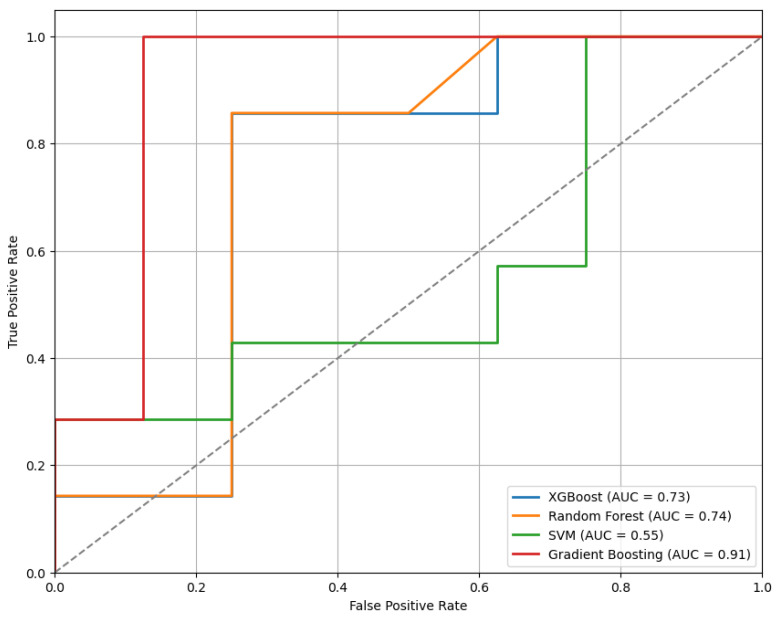
The ROC curves for the models trained on the top 20 features selected by the mRMR method, demonstrating comparative classifier performance.

Figure 5 presents the ROC curves for the models trained using the top 20 features selected by the mRMR method. Among the evaluated classifiers, the GB model demonstrated the best performance, achieving an AUC of 0.91.

#### 3.1.3. mRMR 30 Features

**Figure 6:**
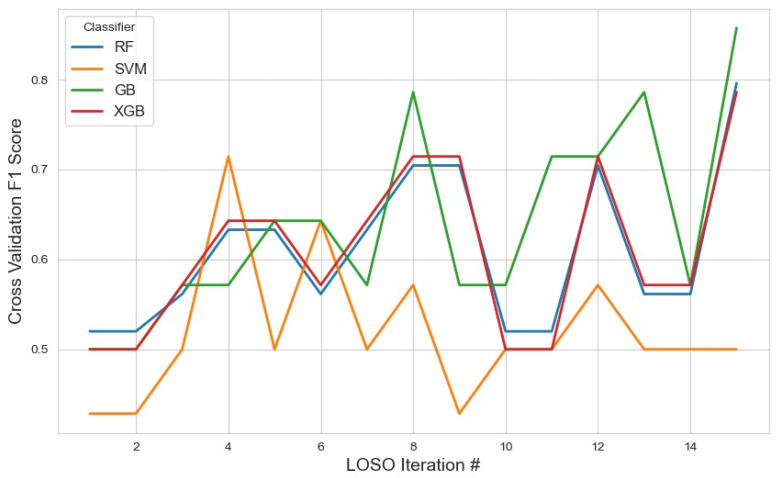
The model performance across iterations using the top 30 features selected by the mRMR method, showing the variation in classification metrics throughout cross-validation and the effect of expanded feature sets on model stability.

Further expanding the selected feature set to 30 features (Figure 6) aimed to evaluate the impact of higher-dimensional input on classification performance while maintaining mRMR’s objective of minimizing redundancy. The complete list of selected features is available in Table 1. In this setup, GB again emerged as the top-performing classifier, with an average F1 score of 0.63 (95% CI: 0.57, 0.69). Although still the best among the models, this performance reflects a slight decline compared to the 10- and 20-feature configurations, suggesting that the inclusion of additional features may have introduced mild redundancy or noise, counterbalancing the potential benefits of increased information. XGB maintained consistent performance with an average F1 score of 0.60, mirroring its results across other feature configurations. In contrast, RF saw a further decline to 0.52, while SVM remained low at 0.51.

**Figure 7:**
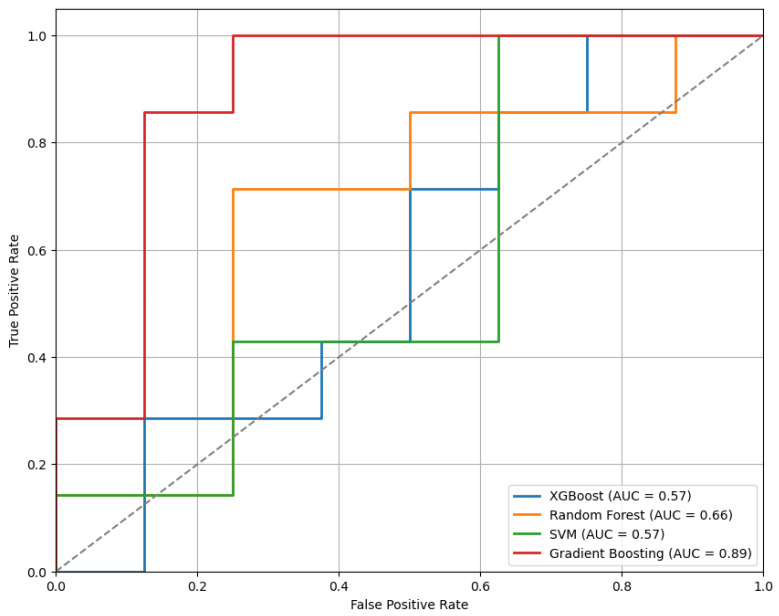
The ROC curves for the models trained on the top 30 features selected by the mRMR method, highlighting differences in classifier performance.

Figure 7 presents the ROC curves for the models trained using the top 30 features selected by the mRMR method. Among the evaluated classifiers, the GB model demonstrated the best performance, achieving an AUC of 0.89.

**Figure 8:**
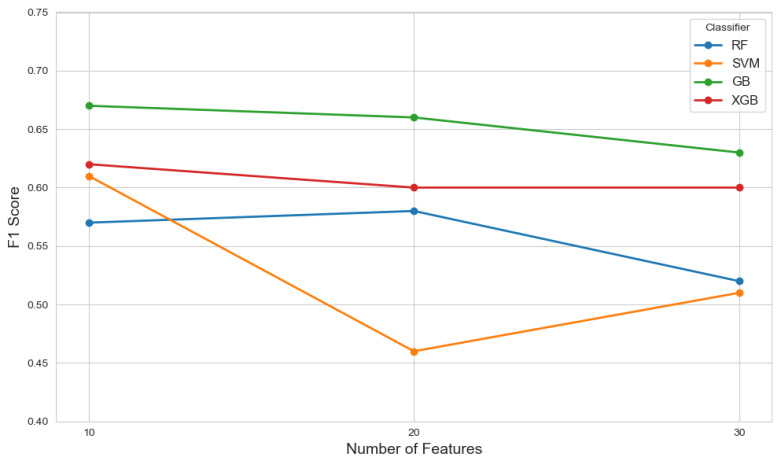
The F1 scores of various classifiers evaluated across different feature set sizes (10, 20, and 30) selected by the mRMR method, illustrating the impact of feature dimensionality on model performance.

The performance of each model across different feature set sizes is illustrated in Figure 8, which presents plots of F1 scores against the number of selected features.

### 3.2. Wrapper-Based Approach

**Figure 9:**
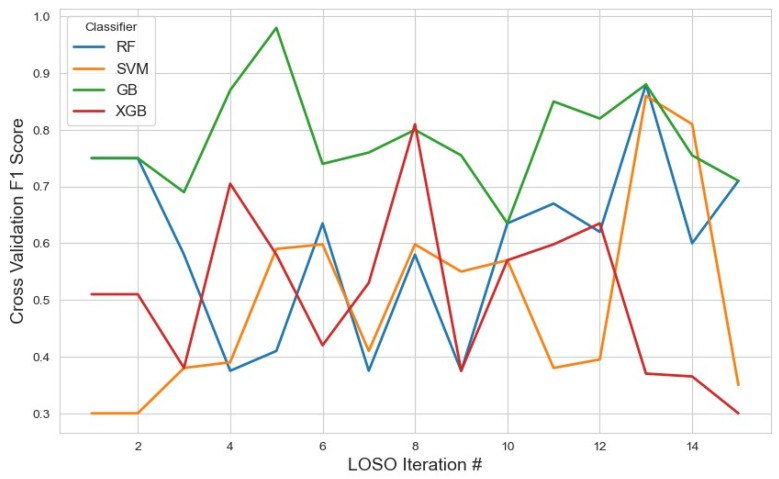
The model performance across iterations using features selected by the RFE method, illustrating classification metric trends during cross-validation and the impact of RFE on model consistency.

For the RFE feature selection method (Figure 9), GB outperformed other classifiers, achieving the highest average F1 score of 0.76 with a 95% confidence interval (CI) of (0.71, 0.82). In contrast, SVM and XGB demonstrated lower performance, with F1 scores of 0.46 and 0.49, respectively. The RF classifier yielded a moderate F1 score of 0.57. These results suggest that GB with RFE was the most effective combination for stress detection, likely due to GB’s iterative nature, which reduces classification errors. With the given feature selection, different classifiers identified varying subsets of features. The list of selected features for each classifier, along with the corresponding results, is summarized in Table 2.

**Figure 10:**
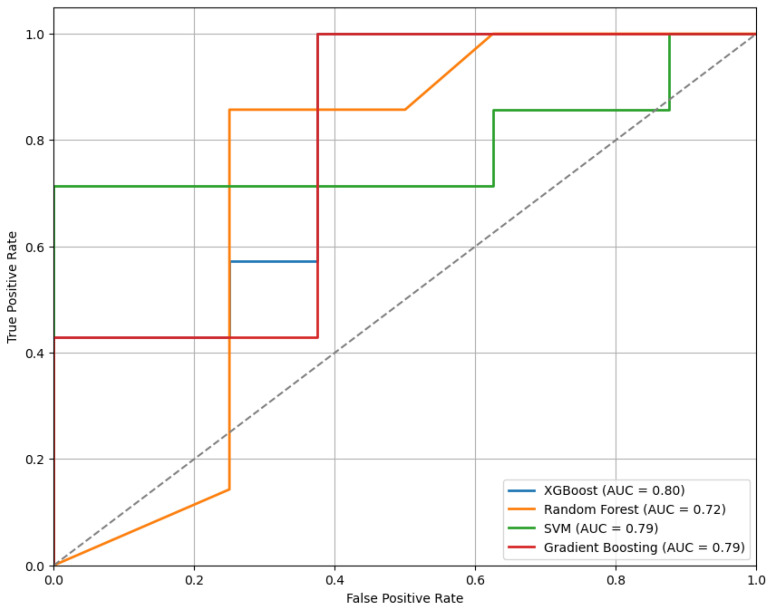
The ROC curves of classifiers trained on features selected via RFE, comparing model performance.

Figure 10 presents the ROC curves for the models trained using the top features selected by the RFE method. Among the evaluated classifiers, the XGBoost model shows the best performance, achieving an AUC of 0.80.

### 3.3. Embedded Approach

**Figure 11:**
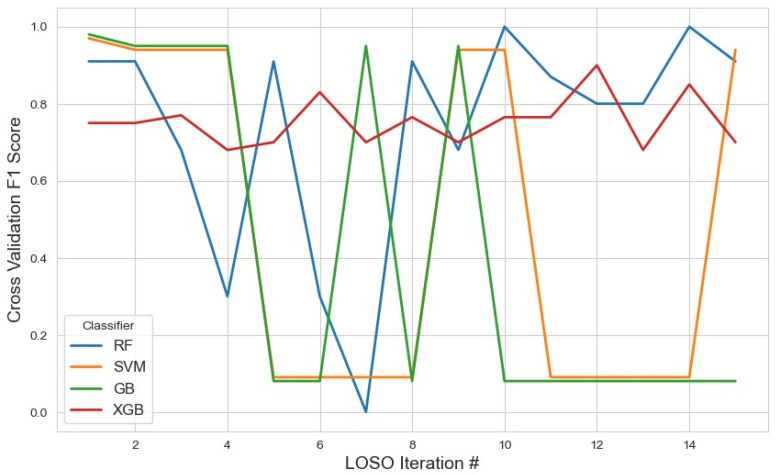
The model performance across iterations using features selected by the LASSO method, highlighting the evolution of classification metrics during cross-validation and the effect of LASSO-based feature selection on model stability.

For the embedded feature selection method using LASSO (Figure 11), the most frequently selected features across iterations were utilized for classification with RF, XGB, SVM, and GB. The results showed that XGB achieved the highest average F1 score of 0.75 (95% CI: 0.71–0.78), slightly outperforming RF, which obtained an average F1 score of 0.73 (95% CI: 0.56–0.90). The stable selection of core features across iterations highlights the robustness of the embedded feature selection method, effectively reducing dimensionality while maintaining strong classification performance. In contrast, SVM achieved a lower average F1 score of 0.46 (95% CI: 0.18–0.74), and GB performed even worse with an F1 score of 0.40 (95% CI: 0.14–0.68). Additionally, the tree-based models (RF, XGB, and GB) were applied separately for feature selection. Among them, XGB demonstrated the highest F1 score of 0.40 (95% CI: 0.12–0.68), followed by GB with 0.33 (95% CI: 0.15–0.61) and RF with 0.20 (95% CI: 0.12–0.42). The detailed list of selected features and complete results for these approaches are summarized in Table 3.

**Figure 12:**
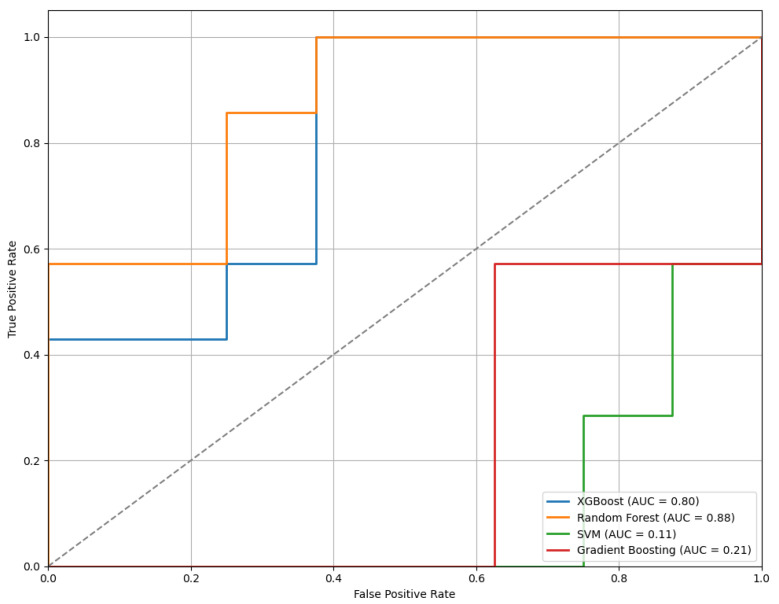
The ROC curves of the models trained on features selected by the LASSO method, illustrating comparative classifier performance.

Figure 12 displays the ROC curves for the models trained using features selected through the LASSO method. Among the classifiers evaluated, the RF model achieved the highest performance with an AUC of 0.88, indicating strong classification capability.

### 3.4. External Validation on Independent Dataset

To evaluate the robustness and generalizability of the proposed framework, external validation was conducted using an independent dataset obtained from PhysioNet. This evaluation employed the best-performing model configuration identified during internal testing—RFE for feature selection combined with GB for classification.

**Figure 13:**
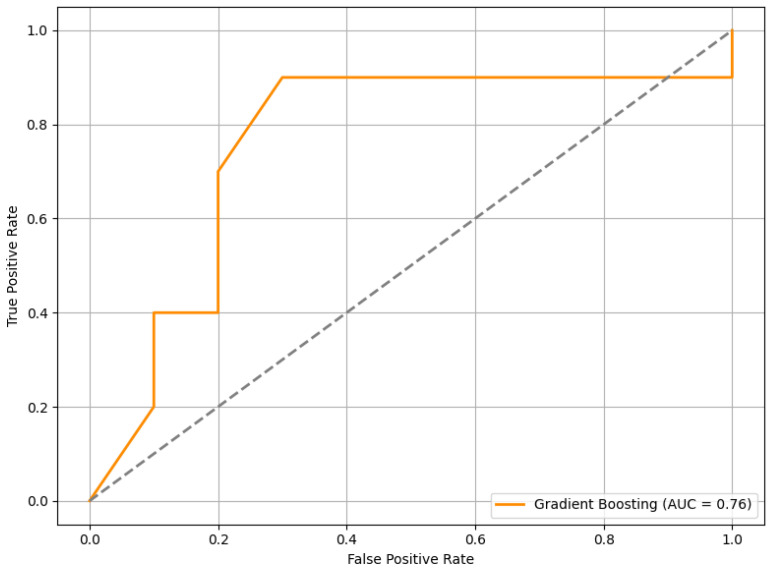
The ROC curve of the RFE+GB model evaluated on the external PhysioNet dataset. The curve illustrates the model’s ability to discriminate between stress and non-stress conditions.

When tested on this completely unseen dataset, the RFE+GB model achieved an average F1 score of 0.63. In addition, we computed the ROC curve, as shown in Figure 13, which yielded an AUC of 0.76. This result indicates a good level of discriminatory power in distinguishing between stress and non-stress conditions.

## 4. Discussion

In this study, we applied three feature selection methods— filter-based, wrapper-based, and embedded—to HRV features extracted from ECG signals to classify stress states. The evaluation was performed using a nested LOSO-CV framework, which ensured that the models were validated on unseen subjects, thus enhancing their generalizability [22]. The primary objective was to identify the most relevant HRV features for distinguishing stress from non-stress states. Out of the 93 HRV features, a small but highly informative subset was selected using the best-performing feature selection approach, RFE combined with GB. Specifically, the median absolute deviation of the RR intervals normalized by the median (HRV-MCVNN) and normalized low frequency power (HRV-LFn) consistently emerged as the most discriminative features. These features played a pivotal role in classifying the stress and non-stress conditions across multiple classifiers, highlighting their physiological relevance in stress response and demonstrating the significance of targeted feature selection in enhancing the performance of machine learning models for stress detection [25].

RR intervals (the time between successive R-peaks in the ECG signal) are often analyzed to understand ANS activity [36]. HRV-MCVNN, the ratio of the median absolute deviation (MAD) of the RR intervals (RRIs) to the median of the RRIs, is a robust derived measure of HRV. The MAD of the RRIs captures the dispersion of RR intervals around the median and is less sensitive to outliers than standard deviation. A high value suggests greater variability in heartbeats, typically reflecting parasympathetic dominance and lower stress. Since HR influences variability, dividing by the median of the RRIs controls for individual or condition-specific HR. This makes comparisons more meaningful across conditions or subjects. During stress, sympathetic activity increases while parasympathetic activity decreases, leading to shorter RRIs, a reduced MAD to the RRIs, and consequently a lower HRV-MCVNN compared to no-stress stress conditions. HRV-MCVNN has been shown to correlate with emotional states, making it a relevant feature for stress detection [37].

The normalized low-frequency (LFn) power of HRV is frequently used as a non-invasive index of ANS function, though its precise physiological interpretation remains subject to ongoing debate [38]. LFn is commonly interpreted as an indicator of sympathetic nervous system activity and of the overall balance between the sympathetic and parasympathetic branches of the autonomic nervous system [39]. The low-frequency (LF) power component (0.04–0.15 Hz) reflects contributions from both sympathetic and parasympathetic influences, whereas the high-frequency (HF) power component (0.15–0.4 Hz) is predominantly mediated by parasympathetic activity and is strongly associated with respiratory sinus arrhythmia and baroreflex function. LFn is computed by expressing LF power as a proportion of the total power within the LF and HF bands. This normalization is intended to provide a more interpretable index of sympathovagal balance, particularly in dynamic contexts, such as stress reactivity. Increases in LFn are traditionally interpreted as reflecting a shift toward sympathetic predominance, whereas decreases in LFn suggest parasympathetic dominance or a more balanced autonomic state. Acute psychological stress typically elicits a shift in autonomic control characterized by increased sympathetic activity and reduced parasympathetic activity, the so-called “fight-or-flight” response [40]. In HRV analyses, this response is commonly reflected in increased LFn values. Conversely, lower LFn values are observed under conditions of relaxation and parasympathetic predominance. Despite its widespread use, the interpretation of LFn as a direct index of sympathetic tone is not universally accepted. The LF band captures complex interactions between autonomic branches, and the relationship between LFn and pure sympathetic activity is neither linear nor exclusive [38]. Nonetheless, an elevated LFn remains a robust and widely reported marker of autonomic shifts toward sympathetic dominance, particularly in the context of stress research. Overall, LFn provides a practical and valuable, albeit imperfect, metric for assessing autonomic nervous system dynamics in response to stressors. While its interpretation should account for the inherent complexity of autonomic regulation, increases in LFn consistently indicate a state of heightened physiological arousal and a shift in autonomic control consistent with the body’s characteristic response to acute stress [41].

The results showed that the combination of RFE and GB achieved the highest average F1 score of 0.76, with a 95% confidence interval of (0.71, 0.82). This is consistent with previous findings where GB’s iterative nature was shown to improve predictive performance by reducing classification errors [31]. The RFE method is known for its ability to eliminate irrelevant features, iteratively enhancing model performance [18]. In contrast, the combination of RFE with other classifiers, such as SVM and XGB, yielded lower F1 scores, highlighting the importance of classifier choice in stress classification models [22].

The mRMR feature selection, which minimizes feature redundancy while maximizing relevance to the target variable, also provided competitive results. For the mRMR (10) feature selection, GB achieved the highest F1 score of 0.67 (95% CI: 0.62–0.72), followed by XGB with score of 0.62 (95% CI: 0.57–0.67). As the number of selected features increased to 20 and 30, the performance of mRMR slightly decreased, with GB’s F1 scores of 0.66 (95% CI: 0.61–0.70) for mRMR (20) and 0.63 (95% CI: 0.57–0.69) for mRMR (30), highlighting that more features may not necessarily lead to improved performance. This trend suggests that, while mRMR effectively reduced redundancy, it struggled to maintain high classification performance when a larger feature subset was used. Our analysis revealed that, while mRMR is effective at identifying informative and non-redundant features, increasing the number of selected features does not always translate to better classification performance. Beyond a certain point, additional features may introduce redundancy or noise, leading to diminishing returns or slight performance declines. This indicates that, although mRMR optimizes feature relevance and redundancy based on mutual information, the selected features may not always align with the classifier’s optimal decision boundary. As a result, tuning the number of features is critical—not only to avoid overfitting, but also to ensure that selected features synergize with the classifier’s decision logic. These findings underscore the importance of tuning feature subset size in tandem with model selection to balance model complexity and generalization performance. Although mRMR did not outperform RFE, which achieved the highest score of 0.76 (95% CI: 0.71–0.82) using GB, it still offered a useful feature subset that captured significant information for stress detection. In comparison, LASSO provided the best overall performance, achieving an F1 score of 0.75 (95% CI: 0.71–0.78) with XGB, demonstrating its ability to produce sparse, interpretable models without sacrificing predictive power [32]. These results underscore the importance of carefully selecting the number of features for optimal performance and highlight the value of mRMR in reducing feature redundancy while maintaining relevance for stress classification.

The findings from this study suggest that only a small subset of HRV features carry the most relevant information for stress classification. The RFE method demonstrated a significant reduction in feature space while maintaining high performance, indicating that iterative feature elimination can effectively reduce dimensionality without compromising classification accuracy. Similarly, the LASSO, by imposing L1 regularization, effectively pruned irrelevant features, leading to a sparse and interpretable model. Although mRMR selected features with strong mutual information with the target variable, it was relatively less effective in reducing redundancy compared to RFE and LASSO, which could explain its slightly lower classification performance.

One of the main challenges in HRV-based stress detection is the high-dimensional nature of the data, which can lead to overfitting if not properly managed. Feature selection methods, like RFE, are crucial in mitigating this issue by reducing the number of features without losing predictive power. The findings from this study are consistent with previous research, which has highlighted the importance of feature selection in improving the efficiency and interpretability of machine learning models applied to physiological signal data [29].

In terms of model validation, the use of nested LOSO-CV was essential for ensuring that our models generalized well to new subjects, as it prevents information leakage and overfitting [22]. This approach is particularly important in applications like stress detection, where individual variability is high, and for ensuring robustness across subjects is critical for real-world deployment.

The external validation results offer insight into the generalizability of the proposed HRV-based stress classification framework. On a completely independent dataset—collected under different experimental conditions, from different participants, and involving varied stress elicitation contexts—the model achieved an F1 score of 0.63 and an AUC of 0.76. While the performance was lower than in internal validation, this outcome was not unexpected given the variability across datasets. Still, the results demonstrate a reasonable level of predictive capability in more diverse conditions, suggesting that the model maintains a useful degree of generalizability beyond the original development setting.

In the original WESAD study [25], the authors achieved an F1 score of 81.31% for stress classification using ECG-derived features, with Linear Discriminant Analysis identified as their best-performing model. However, their feature extraction approach relied on a sliding window technique, resulting in a set of 20 features. In contrast, our study employed the NeuroKit2 package in Python 3.5 for ECG signal processing, which yielded a significantly larger feature set, comprising 93 features. Additionally, we applied feature selection techniques and evaluated alternative classification models to identify the optimal configuration for stress recognition. Our optimal configuration combined GB with RFE, achieving an F1 score of 76%, with a 95% confidence interval ranging from 71% to 82%. While our performance was slightly lower than that reported in [25], it is important to note that the methodological differences—particularly in feature extraction and model selection—may account for the observed variation. These distinctions highlight the sensitivity of model performance to preprocessing and feature engineering choices in physiological stress recognition tasks.

## 5. Limitations and Future Work

This study presents a machine learning framework for stress detection using HRV features; however, several limitations should be acknowledged. First, the limited sample size restricts the statistical power and generalizability of our findings. While we applied techniques, such as LOSO-CV and feature selection, to mitigate overfitting, the dataset remains small relative to the number of features (see Appendix A for univariate feature analysis results). Future studies should involve larger, more diverse cohorts to support robust inference. Second, the small number of female participants may affect HRV patterns and model generalizability. Future work will ensure more balanced representation. Additionally, the feature selection methods identified varying numbers of features due to their inherent selection criteria. To preserve methodological rigor, each method was allowed to operate independently; nonetheless, enforcing a consistent feature count could facilitate more direct comparisons and will be explored in future work. Some models exhibited lower F1 scores, likely reflecting limited sample size, which is now recognized as a study limitation. Although Principal Component Analysis (PCA) was explored, it did not yield improvements over existing feature selection methods and was therefore excluded from the final analysis.

## 6. Conclusions

This study demonstrates the potential of HRV as a reliable biomarker for detecting mental stress through ML techniques, utilizing feature selection methods, like mRMR, RFE, LASSO, and tree-based models, to identify the most relevant HRV features for stress classification. The combination of RFE with GB achieved the best performance, with an F1 score of 0.76, while LASSO created sparse, interpretable models without sacrificing predictive power. HRV-MCVNN and HRV-LFn were the most important features selected to discriminate between stress states. The use of a nested LOSO-CV framework ensured that the models generalized well to unseen subjects, which is essential for real-world applications. This study contributes to the growing body of research on HRV-based stress detection and highlights the importance of effective feature selection in improving the performance of machine learning models. The findings have significant implications for the development of real-time, low-cost stress monitoring systems, with potential applications in workplace health, clinical diagnostics, and wearable health technologies. Future research should explore the integration of additional physiological signals and deep learning models to further enhance stress detection accuracy.

## Figures and Tables

**Table 1:** Performance of the different classifiers with selected mRMR feature sets.

Number of Features	Selected Features	Classifier	F1 Score (95% CI)
10	HRV-MFDFA-alpha2-Width, HRV-pNN50, HRV-Cd, HRV-MFDFA-alpha2-Increment, HRV-MinNN, HRV-Ca, HRV-MSEn, HRV-MFDFA-alpha2-Fluctuation, HRV-MadNN, HRV-C2d	GB	0.67 (0.62, 0.72)
		RF	0.57 (0.52, 0.62)
		SVM	0.61 (0.53, 0.68)
		XGB	0.62 (0.57, 0.67)
20	HRV-MFDFA-alpha2-Width, HRV-pNN50, HRV-Cd, HRV-MFDFA-alpha2-Increment, HRV-MinNN, HRV-Ca, HRV-MSEn, HRV-MFDFA-alpha2-Fluctuation, HRV-MadNN, HRV-C2d, HRV-MFDFA-alpha2-Max, HRV-PAS, HRV-MCVNN, HRV-C2a, HRV-MFDFA-alpha2-Delta, HRV-HTI, HRV-MFDFA-alpha1-Increment, HRV-C1d, HRV-C1a, HRV-SD2a	GB	0.66 (0.61, 0.70)
		RF	0.58 (0.52, 0.63)
		SVM	0.46 (0.40, 0.51)
		XGB	0.60 (0.54, 0.65)
30	HRV-MFDFA-alpha2-Width, HRV-pNN50, HRV-Cd, HRV-MFDFA-alpha2-Increment, HRV-MinNN, HRV-Ca, HRV-MSEn, HRV-MFDFA-alpha2-Fluctuation, HRV-MadNN, HRV-C2d, HRV-MFDFA-alpha2-Max, HRV-PAS, HRV-MCVNN, HRV-C2a, HRV-MFDFA-alpha2-Delta, HRV-HTI, HRV-MFDFA-alpha1-Increment, HRV-C1d, HRV-C1a, HRV-SD2a, HRV-MFDFA-alpha1-Width, HRV-SDNNI1, HRV-PI, HRV-SDNNa, HRV-MFDFA-alpha2-Asymmetry, HRV-SD2, HRV-IQRNN, HRV-S, HRV-SDNN, HRV-SDNNI2	GB	0.63 (0.57, 0.69)
		RF	0.52 (0.46, 0.57)
		SVM	0.51 (0.47, 0.56)
		XGB	0.60 (0.55, 0.66)

**Table 2:** The performance of different classifiers with the wrapper-based feature selection method.

Classifier	Selected Features	Average F1 Score (95% CI)
GB	HRV-MCVNN, HRV-LFn	0.76 (0.71, 0.82)
RF	HRV-MCVNN	0.57 (0.49, 0.66)
SVM	HRV-SDANN1, HRV-SDANN2, HRV-SDNNI2, HRV-MadNN, HRV-pNN20, HRV-MinNN, HRV-SD2d	0.46 (0.38, 0.55)
XGB	HRV-MCVNN, HRV-MSEn	0.49 (0.41, 0.57)

**Table 3:** The performance of different classifiers with embedded feature selection methods.

Feature Selection Method	Selected Features	Classifier	Average F1 Score (95% CI)
LASSO	HRV-MFDFA-alpha2-Width, HRV-MinNN, HRV-LF, HRV-LFn, HRV-MSEn, HRV-MadNN, HRV-Cd	GB	0.40 (0.14, 0.68)
		RF	0.73 (0.56, 0.90)
		SVM	0.46 (0.18, 0.74)
		XGB	0.75 (0.71, 0.78)
Tree-Based	HRV-SD2d, HRV-LF, HRV-SI, HRV-SDNNd, HRV-PSS, HRV-MFDFA-alpha1-Max, HRV-MFDFA-alpha1-Mean, HRV-MFDFA-alpha1-Peak, HRV-MFDFA-alpha1-Width, HRV-MSEn, HRV-PAS, HRV-MCVNN, HRV-C1d, HRV-PIP	GB	0.33 (0.15, 0.61)
		XGB	0.40 (0.12, 0.68)
		RF	0.20 (0.12, 0.42)

## Data Availability

Data are contained within the article or Supplementary Material.

## Supplementary Materials

The following supporting information can be downloaded at: https://www.mdpi.com/article/10.3390/s25134154/s1. Details of the R-peak detection, HRV feature extraction, and machine learning pipeline are provided in the Supplementary Materials (Pseudocode 1–3).

## Abbreviations

The following abbreviations are used in this manuscript [26]:

**Feature**	**Description**
HRV-MeanNN	Mean of RR intervals; reflects overall heart rate and vagal tone.
HRV-SDNN	Standard deviation of RR intervals; indicates total HRV, influenced by both sympathetic and parasympathetic branches.
HRV-SDANN1	Std. dev. of the average RR intervals over 1 min segments; captures long-term HRV components.
HRV-SDNNI1	Mean of std. devs. of RR intervals from 1 min segments; reflects local short-term variability.
HRV-SDANN2	Std. dev. over 2 min segments; sensitive to slower autonomic oscillations.
HRV-SDNNI2	Mean of std. devs from 2 min segments; emphasizes short-term balance.
HRV-SDANN5	Std. dev. over 5 min averages; captures circadian and long-range effects.
HRV-SDNNI5	Mean of 5 min segment variabilities; shows fine-grained autonomic shifts.
HRV-RMSSD	Root mean square of successive RR differences; reliable marker of vagal activity.
HRV-SDSD	Std. dev. of successive RR differences; reflects rapid vagal modulations.
HRV-CVNN	Coefficient of variation of RR intervals; normalized HRV variability.
HRV-CVSD	RMSSD divided by mean RR; normalized index of parasympathetic activity.
HRV-MedianNN	Median RR interval; robust estimate of central heart rhythm.
HRV-MadNN	Median absolute deviation; resilient to outliers, reflects HR stability.
HRV-MCVNN	MAD normalized by median; relative variability index.
HRV-IQRNN	Interquartile range; dispersion of mid-range HRV.
HRV-SDRMSSD	Ratio of SDNN to RMSSD; shows sympathetic–parasympathetic balance.
HRV-Prc20NN	20th percentile; indicative of lower HR bounds.
HRV-Prc80NN	80th percentile; indicative of upper HR bounds.
HRV-pNN50	Percentage of successive RR differences > 50 ms; robust vagal marker.
HRV-pNN20	Percentage of successive RR differences > 20 ms; sensitive to parasympathetic control.
HRV-MinNN	Minimum RR; peaks in sympathetic drive.
HRV-MaxNN	Maximum RR; denotes peak vagal influence.
HRV-HTI	Histogram-based triangular index; overall HRV estimation.
HRV-TINN	Width of RR histogram; reflects total variability.
HRV-ULF	Ultra-low frequency power; linked to circadian and thermoregulatory influences.
HRV-VLF	Very low frequency power; related to slow-acting regulatory systems.
HRV-LF	Low frequency power; reflects both SNS and PNS activity.
HRV-HF	High frequency power; closely tied to respiratory-linked vagal activity.
HRV-VHF	Very high frequency; rarely used, possibly tied to respiratory drive.
HRV-TP	Total spectral power; combined autonomic activity.
HRV-LFHF	LF/HF ratio; widely used indicator of autonomic balance.
HRV-LFn	Normalized LF power; better reflects sympathetic contribution.
HRV-HFn	Normalized HF power; better captures vagal contribution.
HRV-LnHF	Log of HF power; used for statistical normalization.
HRV-SD1	Short-term Poincaré axis; vagal activity.
HRV-SD2	Long-term axis; influenced by both branches of ANS.
HRV-SD1SD2	SD1/SD2 ratio; vagal dominance indicator.
HRV-S	Area of Poincaré ellipse; composite HRV index.
HRV-CSI	SD2/SD1; Cardiac Sympathetic Index.
HRV-CVI	log(SD1 × SD2); Cardiac Vagal Index.
HRV-CSI-Modified	Modified CSI; refined sympathetic activity measure.
HRV-PIP	Percentage of inflection points; nonlinear HRV measure.
HRV-IALS	Inverse average length of segments; fragmentation indicator.
HRV-PSS	Percentage of short segments; instability in autonomic output.
HRV-PAS	Percentage of alternating segments; switching in autonomic tone.
HRV-GI	Guzik Index; vagal-mediated deceleration asymmetry.
HRV-SI	Slope Index; asymmetry slope marker.
HRV-AI	Area Index; captures distributional asymmetry.
HRV-PI	Porta’s Index; general HRV asymmetry.
HRV-C1d/a	Short-term deceleration/acceleration; vagal/sympathetic activity.
HRV-SD1d/a	Short-term deceleration/acceleration variance.
HRV-C2d/a	Long-term deceleration/acceleration contributions.
HRV-SD2d/a	Long-term variability in deceleration/acceleration.
HRV-Cd/a	Total deceleration/acceleration; summarizes vagal/sympathetic effects.
HRV-SDNNd/a	Deceleration/acceleration-related SDNN components.
HRV-DFA-alpha1	Short-term detrended fluctuation; vagal fractal regulation.
HRV-DFA-alpha2	Long-term detrended fluctuation; system complexity.
HRV-MFDFA-alpha1-Width	Multifractal spectrum width.
HRV-MFDFA-alpha1-Peak	Multifractal spectrum peak.
HRV-MFDFA-alpha1-Mean	Mean of multifractal spectrum.
HRV-MFDFA-alpha1-Max	Max of multifractal spectrum.
HRV-MFDFA-alpha1-Min	Min of multifractal spectrum.
HRV-ApEn	Approximate entropy; irregularity of HR series.
HRV-SampEn	Sample entropy; noise-robust HR complexity.
HRV-ShanEn	Shannon entropy; distribution-based variability.
HRV-FuzzyEn	Fuzzy entropy; robust to physiological noise.
HRV-MSEn	Multiscale entropy; reflects system complexity across scales.
HRV-CMSEn	Composite multiscale entropy; reflects system complexity across scales.
HRV-RCMSEn	Refined composite multiscale entropy; reflects system complexity across scales.
HRV-CD	Correlation dimension; fractal ANS dynamics.
HRV-HFD	Higuchi fractal dimension; geometric complexity.
HRV-KFD	Katz fractal dimension; time-series irregularity.
HRV-LZC	Lempel–Ziv complexity; symbolic pattern complexity.

## Appendix A. Univariate Statistical Comparison of HRV Features Between Stress and Non-Stress Conditions

**Table A1:** Univariate analysis results comparing the HRV features between the stress and non-stress conditions.

Feature	Stress (Mean ± SD)	Non-Stress (Mean ± SD)	*p*-Value
HRV-MSEn	1.18 ± 0.11	1.09 ± 0.20	0.15
HRV-MFDFA-alpha2-Delta	−0.21 ± 0.40	−0.52 ± 0.39	0.15
HRV-MFDFA-alpha2-Max	0.55 ± 0.29	0.31 ± 0.32	0.22
HRV-MFDFA-alpha2-Asymmetry	−0.38 ± 0.21	−0.26 ± 0.16	0.22
HRV-MFDFA-alpha2-Width	0.29 ± 0.10	0.45 ± 0.20	0.27
HRV-SDNNI1	74.44 ± 18.05	89.90 ± 30.72	0.27
HRV-MFDFA-alpha2-Increment	0.01 ± 0.00	0.02 ± 0.02	0.27
HRV-C1a	0.43 ± 0.06	0.39 ± 0.05	0.27
HRV-C2d	0.46 ± 0.02	0.44 ± 0.02	0.27
HRV-MFDFA-alpha1-Width	2.19 ± 0.73	1.78 ± 0.62	0.27
HRV-C1d	0.57 ± 0.06	0.61 ± 0.05	0.27
HRV-C2a	0.54 ± 0.02	0.56 ± 0.02	0.27
HRV-CSI-Modified	3571.42 ± 1778.81	4137.19 ± 1640.41	0.27
HRV-MCVNN	0.16 ± 0.03	0.20 ± 0.07	0.27
HRV-Ca	0.53 ± 0.02	0.55 ± 0.02	0.32
HRV-Cd	0.47 ± 0.02	0.45 ± 0.02	0.32
HRV-SDNNI2	80.49 ± 19.61	97.11 ± 33.65	0.32
HRV-SD2	175.25 ± 32.40	212.01 ± 68.82	0.39
HRV-HTI	32.70 ± 8.31	41.75 ± 14.81	0.39
HRV-PAS	0.07 ± 0.03	0.05 ± 0.02	0.39
HRV-MadNN	127.53 ± 28.63	166.92 ± 64.10	0.39
HRV-pNN50	20.95 ± 14.32	26.91 ± 17.15	0.39
HRV-HFn	0.11 ± 0.05	0.12 ± 0.04	0.39
HRV-MFDFA-alpha1-Increment	0.33 ± 0.16	0.25 ± 0.12	0.39
HRV-LFHF	3.44 ± 1.75	2.83 ± 1.12	0.39
HRV-SD2d	118.69 ± 21.11	140.84 ± 44.60	0.45
HRV-SD2a	128.88 ± 24.88	158.40 ± 52.64	0.45
HRV-ShanEn	8.36 ± 0.26	8.57 ± 0.46	0.45
HRV-PI	52.70 ± 2.17	53.87 ± 1.60	0.45
HRV-MFDFA-alpha2-Fluctuation	0.00 ± 0.00	0.00 ± 0.00	0.45
HRV-HFD	1.64 ± 0.11	1.68 ± 0.10	0.45
HRV-CVSD	0.07 ± 0.02	0.08 ± 0.03	0.45
HRV-CVNN	0.16 ± 0.03	0.19 ± 0.05	0.45
HRV-MinNN	356.12 ± 34.64	380.00 ± 52.36	0.45
HRV-SDNNI5	89.91 ± 20.89	107.59 ± 37.38	0.45
HRV-KFD	3.04 ± 0.38	3.26 ± 0.52	0.52
HRV-SDNNd	86.86 ± 15.54	103.40 ± 33.84	0.52
HRV-SDNNa	93.14 ± 17.81	114.03 ± 38.32	0.52
HRV-CVI	5.01 ± 0.21	5.13 ± 0.34	0.52
HRV-S	22,505.91 ± 11,372.80	36,455.01 ± 29,152.34	0.52
HRV-SDANN5	84.46 ± 20.16	100.94 ± 39.80	0.52
HRV-SDANN2	91.26 ± 20.62	108.52 ± 40.81	0.52
HRV-SDANN1	95.58 ± 20.17	114.03 ± 42.12	0.52
HRV-MFDFA-alpha2-Mean	1.09 ± 0.14	1.18 ± 0.19	0.52
HRV-RCMSEn	1.96 ± 0.15	1.96 ± 0.22	0.52
HRV-LFn	0.31 ± 0.09	0.28 ± 0.05	0.52
HRV-MFDFA-alpha1-Delta	1.05 ± 1.40	0.63 ± 1.48	0.52
HRV-IQRNN	181.17 ± 41.47	226.56 ± 87.40	0.52
HRV-SD1d	30.05 ± 11.62	37.56 ± 20.90	0.60
HRV-MFDFA-alpha1-Max	−0.14 ± 1.11	−0.00 ± 1.34	0.60
HRV-MFDFA-alpha2-Peak	1.04 ± 0.08	1.05 ± 0.09	0.60
HRV-pNN20	49.73 ± 15.89	54.06 ± 15.43	0.60
HRV-SDNN	127.37 ± 23.53	153.95 ± 51.04	0.60
HRV-MaxNN	1422.24 ± 245.22	1376.43 ± 217.37	0.64
HRV-SDRMSSD	2.57 ± 1.03	2.54 ± 0.74	0.69
HRV-HF	0.00 ± 0.00	0.00 ± 0.00	0.69
HRV-LnHF	−9.36 ± 1.13	−9.09 ± 1.07	0.69
HRV-AI	50.07 ± 0.09	50.11 ± 0.11	0.69
HRV-SD1SD2	0.23 ± 0.07	0.22 ± 0.06	0.69
HRV-ULF	0.00 ± 0.00	0.00 ± 0.00	0.69
HRV-CSI	5.03 ± 2.09	4.98 ± 1.50	0.69
HRV-VHF	0.00 ± 0.00	0.00 ± 0.00	0.69
HRV-SD1a	25.76 ± 9.57	29.37 ± 14.72	0.77
HRV-CD	1.34 ± 0.14	1.39 ± 0.17	0.77
HRV-MFDFA-alpha1-Fluctuation	0.00 ± 0.00	0.00 ± 0.00	0.77
HRV-PIP	0.42 ± 0.04	0.41 ± 0.05	0.77
HRV-MFDFA-alpha1-Peak	1.60 ± 0.28	1.57 ± 0.33	0.77
HRV-Prc80NN	894.49 ± 98.21	940.71 ± 158.46	0.77
HRV-LZC	0.41 ± 0.13	0.45 ± 0.11	0.77
HRV-IALS	0.41 ± 0.04	0.39 ± 0.05	0.86
HRV-MFDFA-alpha1-Asymmetry	−0.56 ± 0.22	−0.54 ± 0.20	0.86
HRV-RMSSD	56.08 ± 21.01	67.49 ± 36.03	0.86
HRV-PSS	0.64 ± 0.06	0.61 ± 0.09	0.86
HRV-FuzzyEn	0.63 ± 0.18	0.65 ± 0.16	0.86
HRV-SDSD	56.08 ± 21.01	67.49 ± 36.03	0.86
HRV-SD1	39.66 ± 14.86	47.72 ± 25.48	0.86
HRV-MeanNN	785.13 ± 89.89	802.72 ± 121.32	0.86
HRV-MFDFA-alpha1-Mean	1.51 ± 0.31	1.55 ± 0.33	0.86
HRV-CMSEn	1.33 ± 0.07	1.29 ± 0.12	0.86
HRV-ApEn	1.02 ± 0.27	1.05 ± 0.26	0.95
HRV-SampEn	0.84 ± 0.28	0.82 ± 0.31	0.95
HRV-DFA-alpha1	1.29 ± 0.15	1.28 ± 0.18	0.95
HRV-GI	49.99 ± 0.03	50.00 ± 0.04	0.95
HRV-TP	0.00 ± 0.00	0.00 ± 0.00	0.95
HRV-LF	0.00 ± 0.00	0.00 ± 0.00	0.95
HRV-VLF	0.00 ± 0.00	0.00 ± 0.00	0.95
HRV-SI	49.91 ± 0.07	49.91 ± 0.09	1.00
HRV-Prc20NN	668.57 ± 92.24	658.04 ± 106.06	1.00
HRV-MedianNN	791.73 ± 95.18	807.41 ± 129.53	1.00
HRV-DFA-alpha2	1.02 ± 0.07	1.02 ± 0.09	1.00
