# Supplementary material for: Optimizing Mental Stress Detection via Heart Rate Variability Feature Selection
Source: Sensors (Basel). 2025 Jul 3;25(13):4154. doi: 10.3390/s25134154 (PMC12252238; doi:10.3390/s25134154)
Supplement: Supplementary file 1 [file sensors-25-04154-s001.zip › sensors-3648426-supplementary.pdf]

Supplementary Material for

Optimizing Mental Stress Detection via Heart Rate Variability Feature Selection

Pseudocode 1. R-Peak Detection (Pan–Tompkins inspired)

Function detect_r_peaks(ecg_signal, sampling_rate):

1. Apply bandpass filtering to the ECG signal.
2. Compute the derivative of the filtered signal to highlight slope changes.
3. Square the derivative to emphasize large changes and suppress small ones.
4. Perform moving window integration.
5. Identify the local maxima in the integrated signal that exceed a threshold as R-peaks.
6. Return the indices of the detected R-peaks.

Pseudocode 2. HRV Feature Extraction

Function extract_hrv_features(r_peaks, sampling_rate):

1. Use *hrv* library from Neurokit2 package to compute time-domain, frequency-domain, and nonlinear features.
2. Return HRV features.

Pseudocode 3. Nested Leave-One-Subject-Out Cross-Validation

Initialize:

Outer Loop (Leave-One-Subject-Out Cross-Validation):

For each subject:

1. Hold out the current subject's data as the test set.
2. Use the remaining subjects' data as the training set.

Inner Loop (Feature Selection with RFECV):

a. Use Leave-One-Subject-Out on the training data.

b. Initialize a classifier.

c. Apply Recursive Feature Elimination or other feature selection methods with Cross-Validation (RFECV).

d. Identify the optimal subset of features based on F1 score.

e. Store the selected features.

Model Training and Evaluation:

a. Subset the training and test sets using the selected features.

b. Define a hyperparameter search grid for the classifier.

c. Use cross-validated grid search to identify the best hyperparameters.

d. Train the final model using the optimal parameters on the selected features.

e. Predict on the test set.

f. Evaluate performance and record results.
